# Circulating Hsp70: a tumor biomarker for lymph node metastases and early relapse in thoracic cancer

**DOI:** 10.1186/s12885-025-14725-5

**Published:** 2025-08-09

**Authors:** Dominik Lobinger, Nicholas Taylor, Verena Messner, Sophie Seier, Johannes Bodner, Erika Roberts, Ali Bashiri Dezfouli, Alan Graham Pockley, Seyer Safi, Gabriele Multhoff

**Affiliations:** 1 Department of Thoracic Surgery, München Klinik Bogenhausen, Lehrkrankenhaus der Technischen Universität München (TUM), 81925 Munich, Germany; 2 Central Institute for Translational Cancer Research Technische Universität München (TranslaTUM), TUM School of Medicine and Health, Klinikum rechts der Isar, Technische Universität München (TUM), 81675 Munich, Germany; 3 Department of Radiation Oncology, TUM School of Medicine and Health, Klinikum rechts der Isar, Technische Universität München (TUM), 81675 Munich, Germany; 4 Department of Otholaryngology, Head and Neck Surgery, TUM School of Medicine and Health, Klinikum rechts der Isar, Technische Universität München (TUM), 81675 Munich, Germany; 5 John van Geest Cancer Research Centre, School of Science and Technology, Nottingham Trent University, Nottingham, NG11 8NS UK; 6 Division of Thoracic Surgery, University Hospital rechts der Isar, Technische Universität München (TUM), 81675 Munich, Germany

**Keywords:** NSCLC, Extracellular Hsp70, Biomarker, Liquid biopsy, Immune-phenotype

## Abstract

**Background:**

Heat shock protein 70 (Hsp70) which is frequently overexpressed in many different cancer types is also present on the plasma membrane of tumor but not normal cells. The intensity of membrane-expressed Hsp70 (mHsp70) is associated with disease progression and treatment resistance. It has also been shown that Hsp70 can be actively released into the circulation by mHsp70 positive, viable tumor cells in the form of extracellular lipid microvesicles expressing mHsp70, the levels of which might therefore act as a potential biomarker for tumor aggressiveness in lung malignancies.

**Methods:**

Extracellular Hsp70 (eHsp70) was measured in the plasma of patients with non-small cell lung cancer (*n* = 178, NSCLC) and lung metastases of extrathoracic tumors (*n* = 35) prior to surgery using the Hsp70-exo ELISA which detects microvesicle-associated eHsp70 and the patient`s immunophenotype was determined by flow cytometric analysis of the corresponding peripheral blood lymphocytes.

**Results:**

eHsp70 values were significantly higher in patients with NSCLC than in healthy individuals, with no differences between adeno and squamous cell carcinomas. Levels of circulating eHsp70 which are associated with the Programmed cell death protein 1 (PD-L1) status, gradually increased from early stage to metastatic disease, and patients with lymph node metastases in surgically treatable NSCLC had significantly higher eHsp70 levels than nodal negative patients. In all tumor stages, total lymphocyte counts were significantly reduced and immunoregulatory T (Treg) cell counts were increased compared to healthy controls. Lower CD4 + T helper cell and higher CD3-/CD56+/CD94+/CD69+/NKp30+/NKp46 + NK cell ratios were only found in patients with thoracic metastases of other primary tumors. An early relapse after complete resection with curative intent correlated with significantly elevated eHsp70 levels which were measured prior to surgery, in all thoracic cancer patients.

**Conclusions:**

In summary, we propose circulating eHsp70 levels before any treatment as a predictive biomarker for the presence of lymph node metastases and early therapy failure in patients with thoracic malignancies.

**Supplementary Information:**

The online version contains supplementary material available at 10.1186/s12885-025-14725-5.

## Background

Non-small cell lung cancer (NSCLC), which accounts for approximately 85% of all lung cancer cases, continues to be the cancer with the highest mortality rate worldwide [1]. With 22,590 female and 34,100 male cases registered by the Robert Koch Institute in Germany in 2020, lung cancer remains among the most frequently newly diagnosed types of cancer in the last decade [2]. Upon initial presentation, about two thirds of patients are diagnosed with metastatic NSCLC [3, 4]. Survival rates are heavily influenced by the tumor stage, which besides tumor size, is determined by the presence and extent of lymphatic and distant metastases [5, 6]. Only a minority of patients with early-stage NSCLC for whom complete resection with curative intent with systematic lymph node dissection is the standard treatment have a favourable outcome, whereas most patients with locally advanced and metastatic NSCLC show an unfavourable prognosis despite multimodal therapies [7–9].

Low-dose computed tomography (LDCT) screening can increase the proportion of cases diagnosed in earlier tumor stages and thereby might reduce lung cancer mortality [10]. Despite a high sensitivity for the detection of pulmonary lesions, the specificity of LDCT in differentiating a pulmonary lesion in malignant or benign disease is limited. Therefore, the identification of a tumor biomarker which is able to early determine tumor aggressiveness, especially given the rapidly growing numbers of neoadjuvant and perioperative treatment options [8, 9], is urgently needed. With this in mind, we investigated the potential of circulating extracellular heat shock protein 70 (eHsp70), together with the immunophenotype of peripheral blood lymphocytes (PBLs), as a potential tumor biomarker approach in thoracic oncology.

Intracellularly, Hsp70 plays an important role in maintaining protein homeostasis in all cell types [11–13]. In malignant cells, Hsp70 is often highly overexpressed and presented on the plasma membrane in a tumor-specific manner [14]. Membrane Hsp70 (mHsp70) positive tumor cells actively release Hsp70 in the context of extracellular lipid microvesicles [15]. Depending on the localization of Hsp70 in tumor cells, its role can be contradictory: whereas elevated intracellular levels are associated with an increased resistance to chemo- or radiotherapy [16], membrane-bound and circulating extracellular Hsp70 (eHsp70) can mediate immunomodulatory effects on T and NK cells. Previous studies have only explored free circulating Hsp70 [17, 18], whereas we have developed the Hsp70-exo sandwich Enzyme-linked immunosorbent assay (ELISA) which is able to measure not only free Hsp70 derived from dying cells, but also eHsp70 that is actively released from living tumor cells in lipid microvesicles [19–22].

The aim of this study was to investigate the potential value of eHsp70 levels, as measured using the Hsp70-exo ELISA, and the peripheral blood immunophenotype, as determined by multiparameter flow cytometry, as biomarkers collected before start of any treatment for estimating the likelihood of a lung lesion being malignant and assessing disease aggressiveness in thoracic malignancies.

## Methods

### Patient cohort

**Fig. 1:**
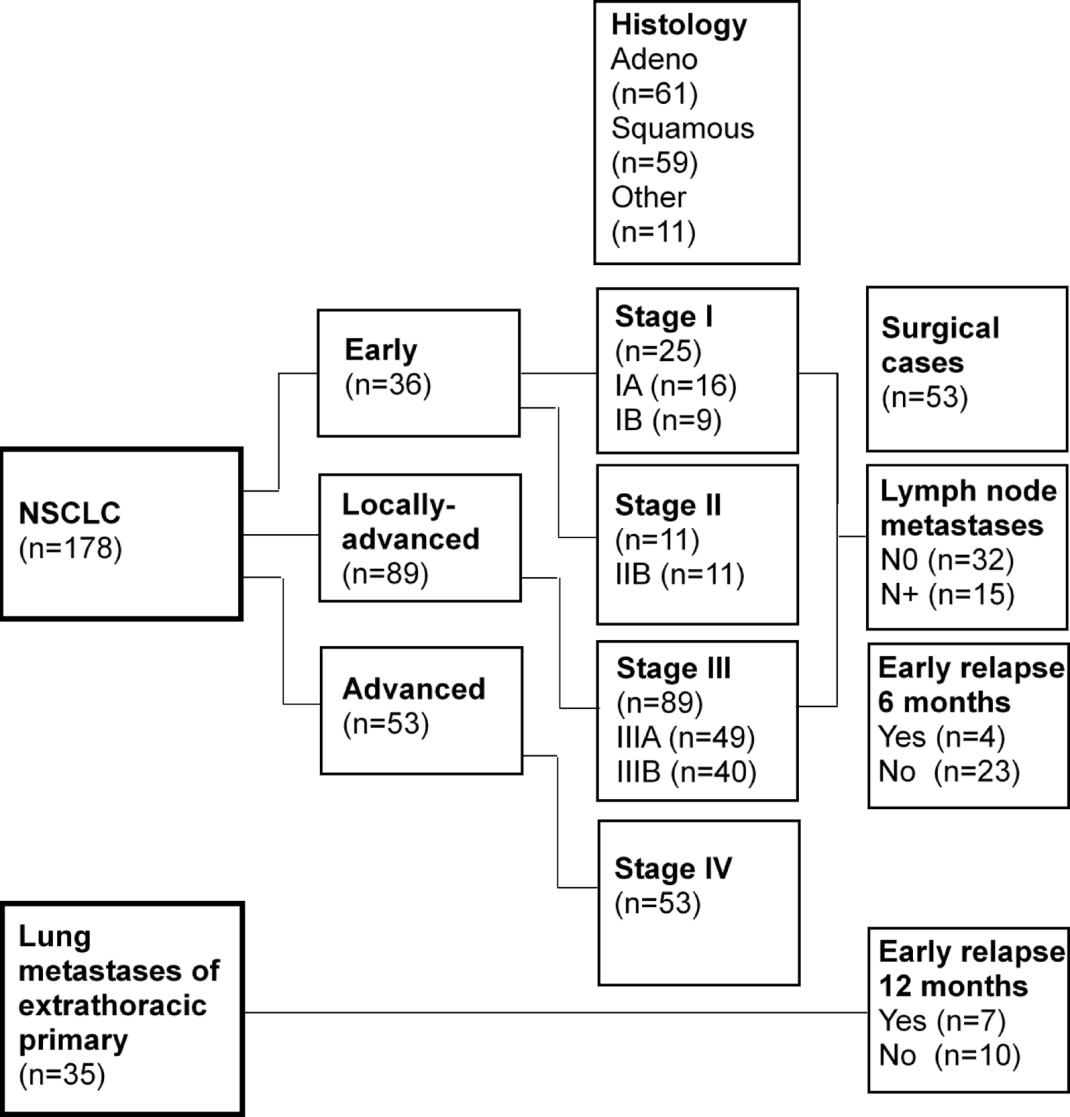
Flow diagram summarizing characteristics (tumor stage, histology, lymph node status, early relapse) of patients with non-small cell lung cancer (NSCLC) in different stages and lung metastases of extrathoracic primary tumors included in this study

The patient cohort consisted of a total of 178 patients diagnosed with NSCLC (*n* = 178) and 35 patients with lung metastases (*n* = 35) of extrathoracic primary tumors (Fig. 1). Among the NSCLC patients, 53 (in total 23 female and 30 male; [n] stage I– 25, II– 11, III– 17, overview with median age in Supplementary Table 1A) had undergone surgical resection with curative intent between 2020 and 2023 at the University Hospital of the Technical University of Munich (TUM) and at the München Klinik Bogenhausen, an academic teaching hospital of TUM. Tumor, nodal and metastatic (TNM) stage and Union for International Cancer Control (UICC)-classification of the surgical cases are summarized in Supplementary Table 1B. The male to female ratio of the patients was 1.3 to 1. Samples from 108 age-matched healthy donors (age range 21–77 years) were additionally included in the analyses. Patient characteristics are summarized in the flow diagram (Fig. 1). All ethylenediamide tetraacetic acid (EDTA) blood samples for eHsp70 analysis were collected before surgery. In patients with lung metastases, blood was drawn once prior to their pulmonary metastasectomy (9 female and 26 male patients, median age 64 years). The study was approved by the local ethics committee of the TUM School of Medicine and Health (ethical approval number: 2403/09 and 2428/09), and written informed consent was obtained from all patients and healthy donors before start of the study. The study was conducted in accordance with the guidelines of the Declaration of Helsinki.

### Hsp70-exo sandwich ELISA

Plasma derived from EDTA anti-coagulated blood (S-Monovette, Sarstedt, Nümbrecht, Germany) was prepared by a centrifugation at 1500xg for 15 min at room temperature, followed by aliquoting (300 µL) and storage of the plasma samples at − 80 °C. The Hsp70-exo sandwich ELISA was performed in 96-well MaxiSorp Nunc-Immuno plates (Thermo, Rochester, NY, USA) using the cmHsp70.2 coating monoclonal antibody (mAb, 1 µg/mL) and the biotinylated cmHsp70.1 mAb (200 ng/mL; multimmune GmbH, Munich, Germany) dissolved in horseradish peroxidase (HRP)-Protector™ (Candor Bioscience GmbH, Wangen i. Allgäu, Germany) as detection agent [19]. Briefly, plasma samples (100 µL) and the Hsp70 protein standard (1-100 ng/mL) were diluted in StabilZyme Select (1:5; Diarect GmbH, Freiburg i. Breisgau, Germany). After a 30 min incubation period at room temperature and another washing, plates were incubated with Streptavidin (57 ng/mL, 30 min; Senova GmbH, Weimar, Germany) in HRP-Protector™ (Candor Bioscience GmbH, Wangern i. Allgäu, Germany). Colorimetric analysis was performed after adding the substrate reagent (100 µL, 15 min; BioFX TMB Super Sensitive One Component HRP Microwell Substrate, Surmodics, Inc., Eden Prairie, MN, USA). After quenching the reaction by adding 2 N H_2_SO_4_ (50 µL), the absorbance was read at 450 nm using a VICTOR X4 Multilabel Plate Reader (PerkinElmer, Waltham, MA, USA), after a correction of the reference absorbance at 570 nm.

### Multiparameter flow cytometry of the immunophenotype in EDTA blood

Freshly taken anti-coagulated EDTA blood (100 µL) was immunophenotyped using a BD FACSCalibur™ flow cytometer (BD Biosciences, Heidelberg, Germany) and the following fluorescence antibody combinations. Combining four different fluorescence-labelled antibodies (fluorescein isothiocyanate (FITC)/phycoerythrin (PE)/peridinin chlorophyll A protein (PerCP)/allophycocyanine (APC)) allowed the following lymphocyte subpopulations to be determined: CD45 + lymphocytes, CD3-/CD19 + B cells, CD3+/CD45 + T cells, CD3+/CD4 + helper T cells, CD3+/CD8 + cytotoxic T cells, CD3+/CD69 + activated T cells, CD3+/CD4+/CD25+/FoxP3 + and CD3+/CD8+/CD25+/FoxP3 + immunoregulatory T cells (Treg), CD3+/CD56 + natural killer (NK)-like T cells (NKT), CD3+/CD94 + NKT cells, CD3+/CD56+/CD69 + NKT, CD3+/NKG2D + NKT, CD3-/CD56 + natural killer (NK) cells, CD3-/CD94 + NK cells, CD3-/CD56+/CD69 + activated NK cells, CD3-/NKG2D + NK cells, CD3-/NKp30 + NK cells, CD3-/NKp46 + NK cells. The antibody combinations and fluorescence dyes used in this study have been described previously [23]. Isotype-matched control antibodies with the respective dyes were used as negative controls. The percentage of a certain lymphocyte subpopulation is defined as the proportion of cells stained positively for a specific antibody within a defined viable lymphocyte gate.

### Statistical analysis

Comparisons among different groups were conducted using ANOVA and post-hoc Tukey tests, and non-parametric data were analysed using the Kruskal Wallis test, as appropriate. The Pearson correlation test was used to determine associations between largest tumor diameter (cm) of the surgically resected tumors and eHsp70 levels. Normal distribution was tested by the Shapiro Wilk normality test. Statistical analysis was performed using the programming language R (R studio version 2024.04.2 + 764). Differences are considered to be statistically significant as follows: not significant, *p < 0.05, **p < 0.01, ***p < 0.0001, ****p < 0.00001.

## Results

### Patient characteristics

NSCLC patients (*n* = 178) were classified into the following disease groups: early (*n* = 36, stages I and II), locally advanced (*n* = 89, stage III) and advanced (*n* = 53, stage IV) (Fig. 1). The flow diagram (Fig. 1) shows that 25 of the 178 NSCLC patients were diagnosed with stage I disease (*n* = 25, stage I; *n* = 16, stage IA; *n* = 9, stage IB), 11 with stage II disease (*n* = 11, stage II), 89 with stage III disease (*n* = 89, stage III; *n* = 49, stage IIIA; *n* = 40, stage IIIB), and 53 with stage IV disease (*n* = 53, stage IV). Sixty-one NSCLC patients had an adeno (*n* = 61, adeno) and 59 a squamous cell carcinoma (*n* = 59, squamous) (Fig. 1). Of the patients with surgically treated NSCLC (*n* = 53), 32 had no lymph node metastases (*n* = 32, N0) and 15 had lymph node metastases (*n* = 15, N+). An early relapse was determined in 4 NSCLC patients (*n* = 23, no early relapse) after 6 months, and in 7 patients with lung metastases of extrathoracic primary tumors (*n* = 10, no early relapse) after 12 months (Fig. 1).

### Circulating eHsp70 concentrations in patients with adeno and squamous cell NSCLC compared to healthy controls

**Fig. 2:**
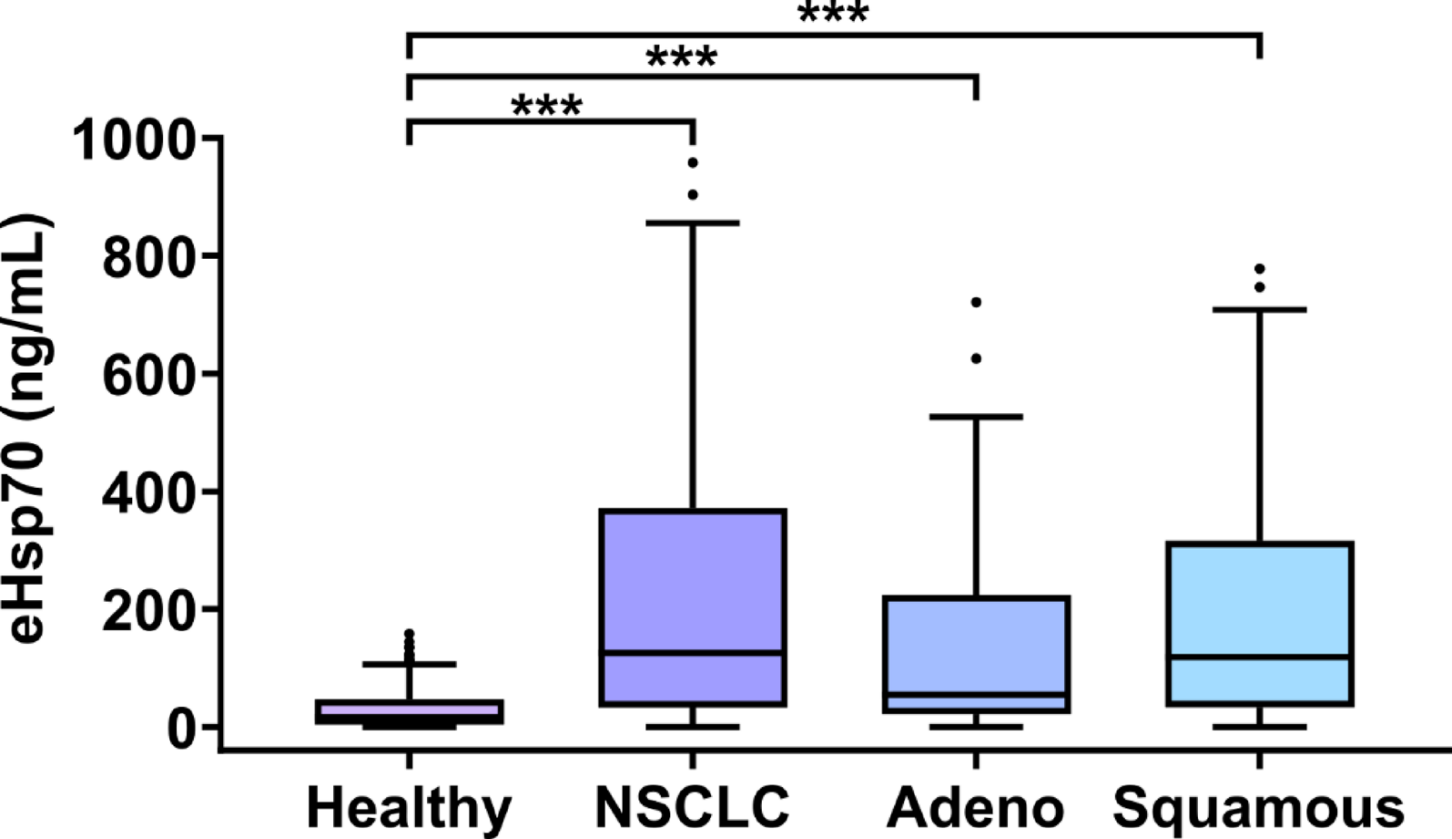
Free and vesicular eHsp70 (ng/mL) levels measured in the plasma of healthy individuals (*n* = 108, Healthy) and NSCLC patients (*n* = 178, NSCLC) with adeno (*n* = 61, adeno) and squamous cell (*n* = 59, Squamous) carcinoma subtypes. Statistically significant differences ***p < 0.001

A comparison of circulating eHsp70 levels measured with the Hsp70-exo ELISA in all NSCLC patients across all four tumor stages prior to the start of any treatment (*n* = 178, median 125.4 ng/mL; ***p < 0.001) demonstrated significantly higher plasma levels of eHsp70 in the tumor patients compared to the healthy control group (*n* = 108, median 16.4 ng/mL), as illustrated in Fig. 2. No difference in eHsp70 levels was found between patients with adeno (*n* = 61, adeno) or squamous cell carcinoma (*n* = 59, squamous). Patients with adeno and squamous cell carcinoma showed significantly higher eHsp70 values in the circulation (***p < 0.001) than the control group (Fig. 2). With respect to the peripheral blood immunophenotype, no significant differences were observed in the lymphocyte subpopulations of adeno and squamous cell carcinoma, in comparison to a healthy control group significant differences were observed predominantly to squamous cell carcinoma patients with respect to CD3+/CD4+/CD8 + T cells, CD3+/CD4+/CD8+/FoxP3 + Treg cells, CD3+/CD56+/CD94+/NKG2D + NKT cells and CD3-/CD56+/CD69 + NK cells (Supplementary Fig. 1A–O). Patients with carcinoma precursors such as lepidic carcinoma (*n* = 2, median 15.5 ng/mL) and low-grade neuroendocrine tumors such as typical carcinoids (*n* = 2, median 7.7 ng/mL) exhibited considerably lower eHsp70 values than patients with advanced NSCLC.

### Circulating eHsp70 concentrations in patients with early, locally advanced and advanced NSCLC

**Fig. 3:**
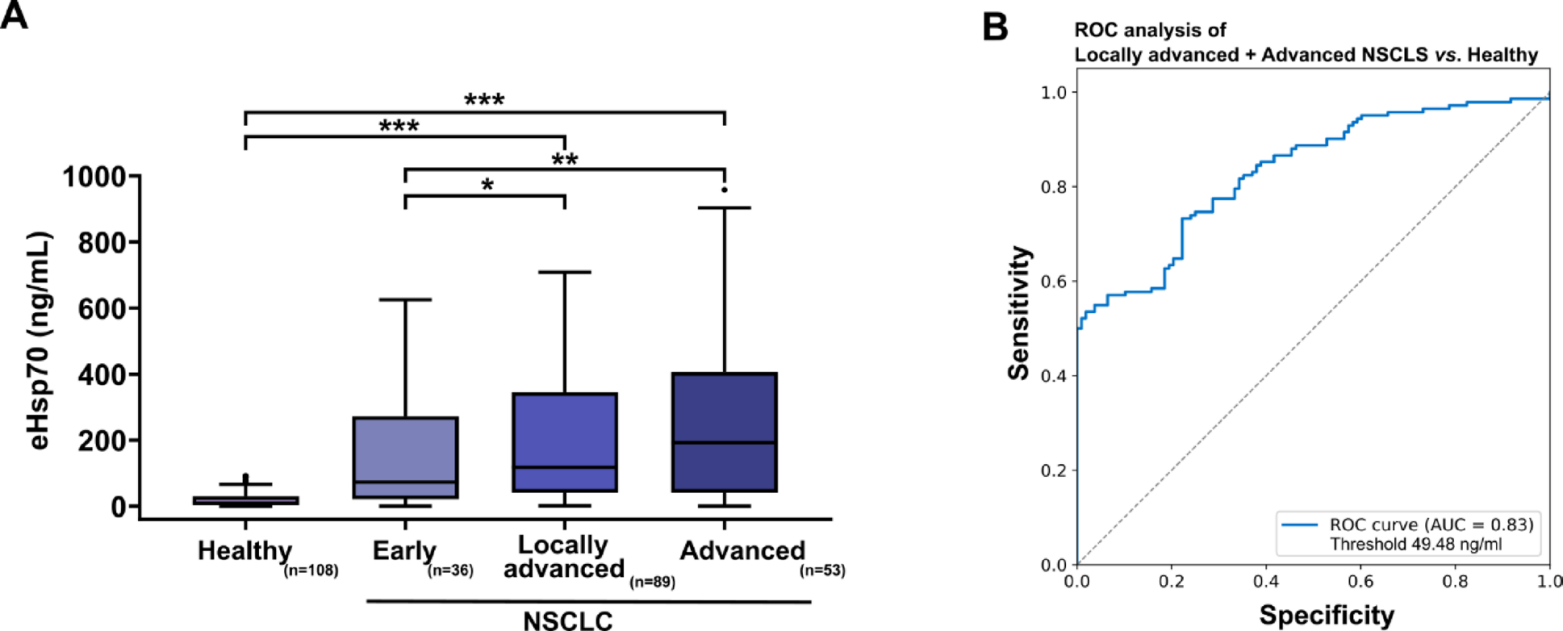
**A** Free and vesicular eHsp70 (ng/mL) levels measured in the plasma of healthy individuals (*n* = 108, Healthy), and patients with early (*n* = 36, Early), locally advanced (*n* = 89, Locally advanced) and advanced (*n* = 53, Advanced) NSCLC. Statistically significant differences *p < 0.05, **p < 0.01, ***p < 0.001. **B** ROC analysis illustrating the sensitivity and specificity of eHsp70 values in patients with NSCLC in locally advanced and advanced stages III and IV compared to healthy donors: AUC value: 0.83, sensitivity: 0.73, specificity: 0.78 at an optimal threshold value of 49.48 ng/mL (“closest top left method”)

Circulating eHsp70 levels increased steadily from early (*n* = 36, stage I and II, median 37.35 ng/mL) through locally advanced (*n* = 89, stage III, median 137.2 ng/mL) to advanced stage with distant metastases (*n* = 53, stage IV, median 199.5 ng/mL), as shown in Fig. 3A. Significant different levels were found between the healthy control group (*n* = 108, median 16.4 ng/mL) and patients with locally advanced tumors and advanced tumors (***p= 0.001, each),  as well as between early stages and locally advanced (*p = 0.05) and advanced NSCLC cases (**p =.01). A receiver operating characteristic (ROC) analysis of circulating eHsp70 levels in locally advanced (stage III) and advanced (stage IV) NSCLC patients compared to healthy donors yielded an area under the curve (AUC) value of 0.83, a sensitivity of 0.73 and a specificity of 0.78 at an optimal threshold value of 49.48 ng/mL (“closest top left method”) (Fig. 3B).

A comparison of the eHsp70 values in the tumor stages I (*n* = 25, median 27 ng/mL), II (*n* = 11, median 287.4 ng/mL), III (*n* = 89, median 137.2 ng/mL) and IV (*n* = 53, median 199.5 ng/mL) revealed significant differences between the control group (median 16.4 ng/mL) and stage III and IV tumors (***p< 0.001, each ), as well as stage I tumors and stage III and IV tumors (**p< 0.01, each) (Supplementary Fig. 2A). A more detailed breakdown confirms the trend described before, with significant differences between the healthy donors and stage IIIA (*n* = 49, median 165.3 ng/mL), IIIB (*n* = 40, median 88.1 ng/mL) and IV (*n* = 53, median 199.5 ng/mL, ***p< 0.001, each ) NSCLC. A noticeable distinction was observed between stage IA (*n* = 16, median 26.4 ng/mL) and stages IIIA and IV disease (each *p< 0.05,) (Supplementary Fig. 2B).

A correlation of the eHsp70 values with the largest pathologically measured NSCLC diameter after surgery showed no obvious correlation (*n* = 47, R^2^ = 0.0616, mean diameter 3.26 cm, Pearson correlation test *p* = 0.25) (Supplementary Fig. 3).

### Circulating eHsp70 concentrations in NSCLC patients with and without lymph node metastases

**Fig. 4:**
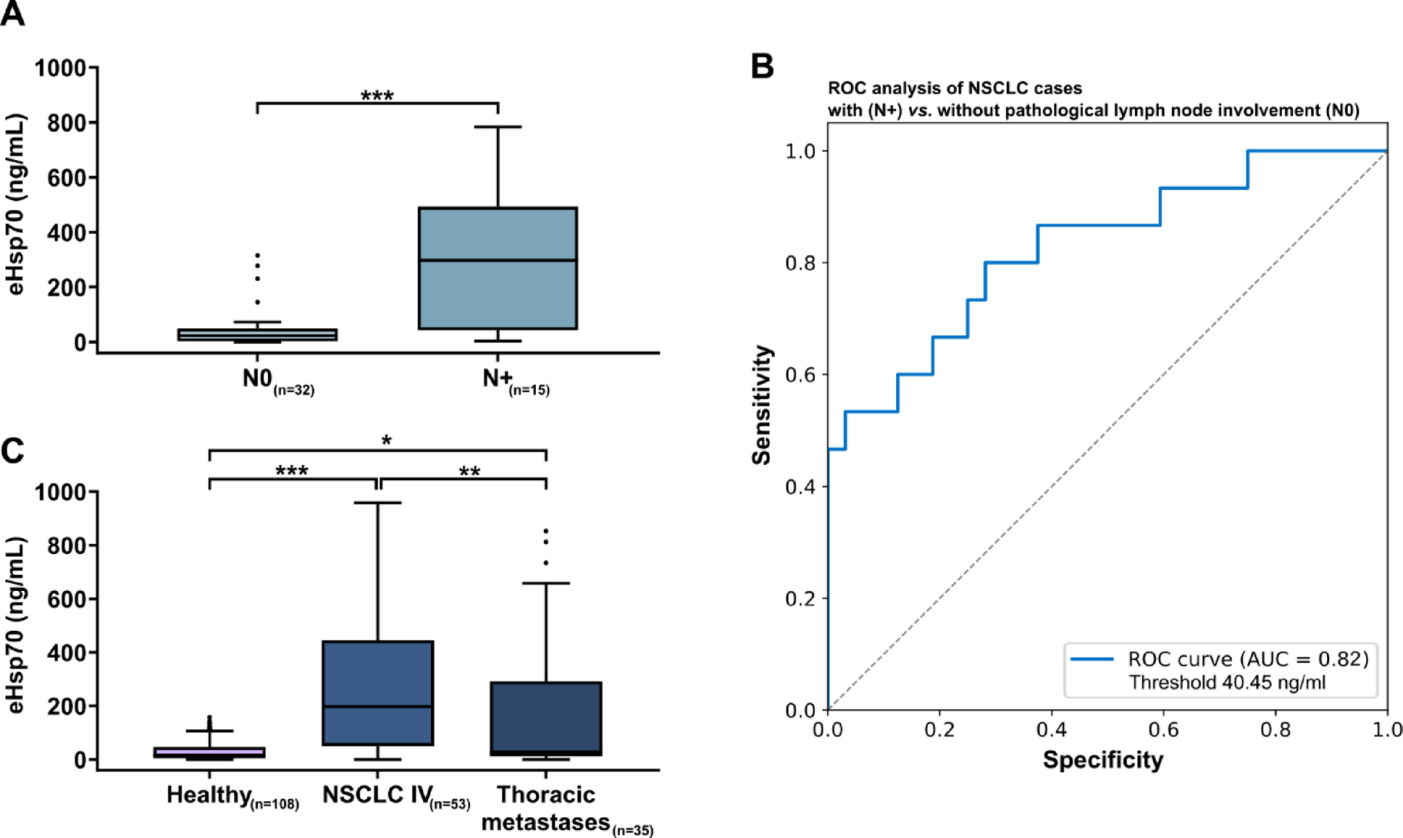
**A** Free and vesicular eHsp70 (ng/mL) levels measured in the plasma of NSCLC patients without (*n* = 32, N0) and with (*n* = 15, N+) pathologically proven lymph node metastases. Statistically significant differences ***p < 0.001. **B** ROC analysis illustrating the sensitivity and specificity of eHsp70 values in NSCLC patients with and without pathological proven lymph node metastases. AUC value: 0.82, sensitivity: 0.8, specificity: 0.71 at an optimal threshold value of 40.45 ng/mL (“closest top left method”). **C** Free and vesicular eHsp70 (ng/mL) levels measured in the plasma of healthy individuals (*n* = 108, Healthy), stage IV NSCLC patients (*n* = 53, NSCLC IV), and patients with lung metastases (*n* = 35, Thoracic metastases) of extrathoracic primary tumors. Statistically significant differences *p < 0.05, **p < 0.01, ***p < 0.001

In NSCLC patients that underwent surgery with curative intent, significantly higher eHsp70 values (***p < 0.001) were present in the subgroup of patients with lymph node metastases (*n* = 15, median 297.2 ng/mL), compared to those with tumor-free lymph nodes (*n* = 32, median 23.34 ng/mL) (Fig. 4A). The ROC analysis of those cases with and without pathological lymph node involvement obtained an AUC value of 0.82 with an achieved sensitivity of 0.8 and a specificity of 0.71 for the prediction of lymph node involvement at an optimal threshold value of 40.45 ng/mL (“closest top left method”), (Fig. 4B). In patients with lymph node metastases, increased eHsp70 levels (*n* = 15, median 297.2 ng/mL) were predominantly found in higher tumor grades: (G3 or micropapillary/solid in 40%; G2 or acinar/papillary in 33%; G1 or lepidic in 0%; other in 26.67%), whereas in patients without lymphatic involvement low eHsp70 levels (*n* = 32, median 23.81 ng/mL) were predominantly found in lower tumor grades (G3 or micropapillary/solid in 29%; G2 or acinar/papillary in 41.9%; G1 or lepidic in 19.4%; other in 9.7%). PD-L1 expression, as determined by immunohistochemistry, was associated with lower eHsp70 levels. In patients with PD-L1 negative tumors (*n* = 17), the median eHsp70 level was significantly higher (277.7 ng/mL) than in those with PD-L1 positive tumors (*n* = 12), with a median eHsp70 level of 21.21 ng/mL (*p < 0.05*). These data indicate that eHsp70 levels might be predictive for the immunotherapy response towards PD-L1 inhibition. However, future studies with larger patient cohorts are necessary to confirm this association.

### Circulating eHsp70 concentrations and peripheral blood immunophenotype in lung cancer patients

**Fig. 5:**
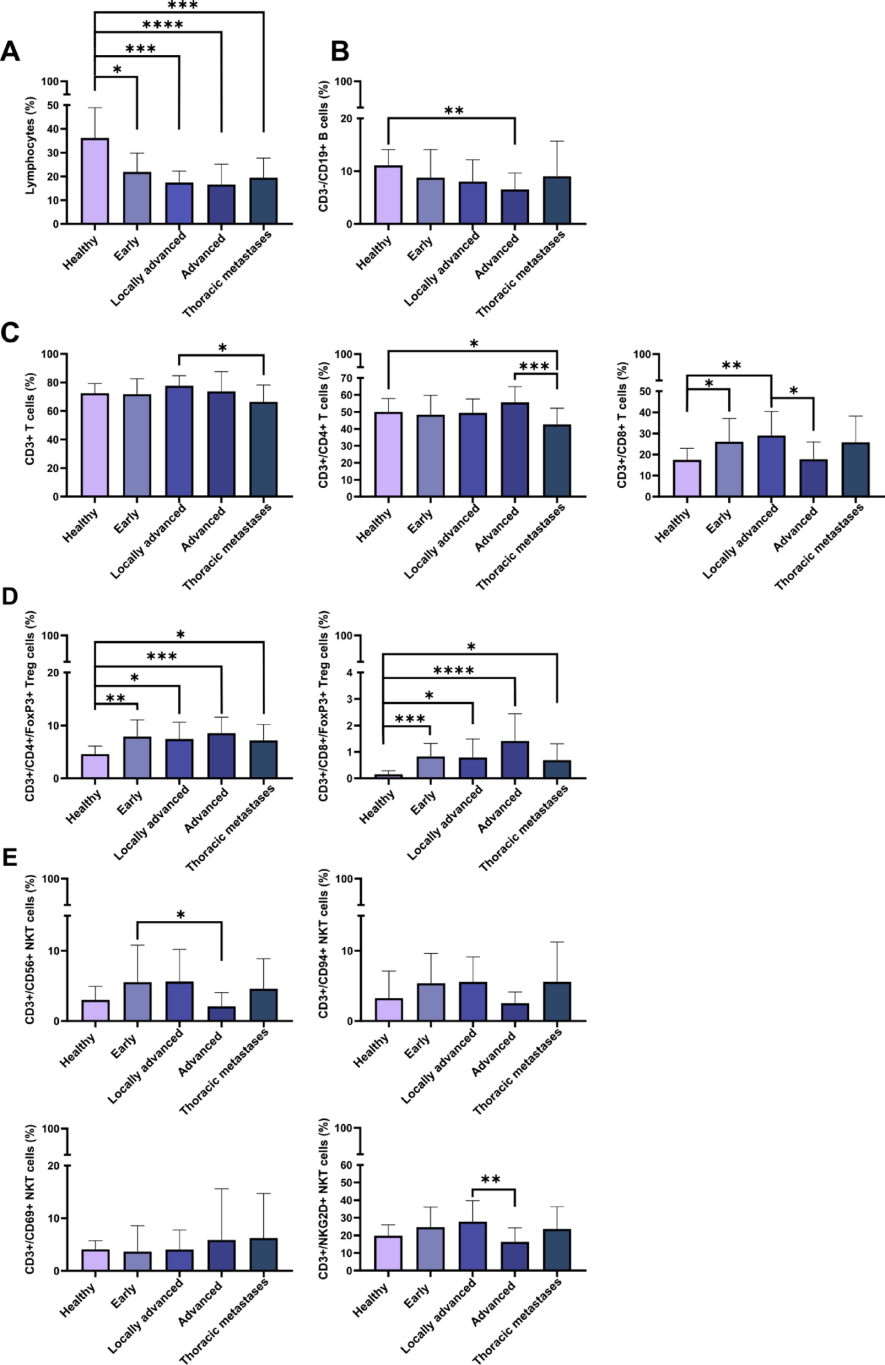

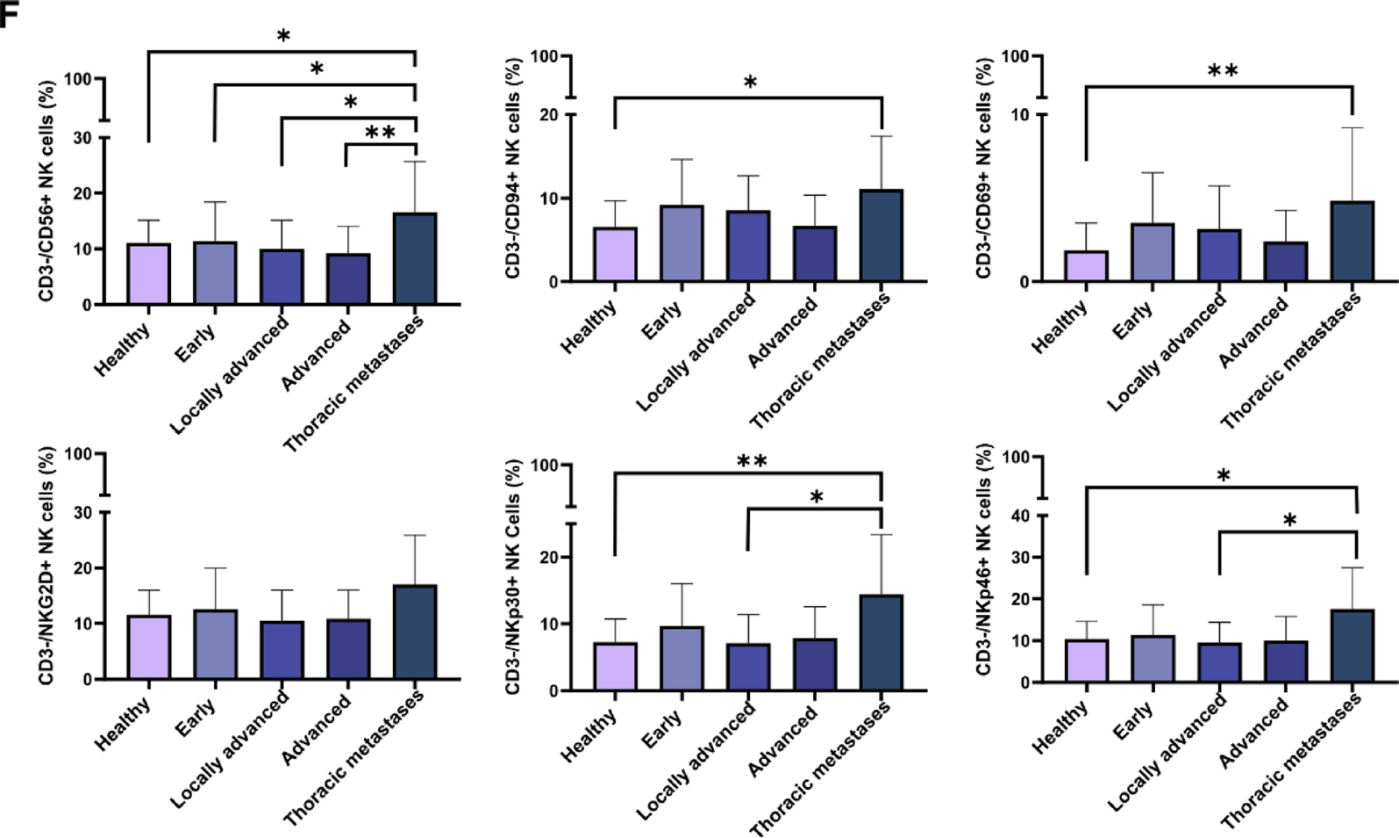
Proportions of lymphocyte subsets in the peripheral blood of healthy donors (*n* = 32), NSCLC patients with early (*n* = 26), locally advanced (*n* = 26), advanced (*n* = 15) tumors, and patients with lung metastases (Thoracic metastases) of extrathoracic primary tumors (*n* = 25). **A**CD45 + lymphocyte counts, **B** CD3-/CD19 + B cells, **C** CD3 + T cells, CD3+/CD4 + T helper cells, CD3+/CD8 + cytotoxic T cells, **D**CD3+/CD4+/FoxP3 + Treg cells, CD3+/CD8+/FoxP3 + Treg cells, **E** CD3+/CD56 + NKT cells, CD3+/CD56+/CD94 + NKT cells, CD3+/CD56+/CD69 + NKT cells, CD3+/CD56+/NKG2D + NKT cells, **F** CD3-/CD56 + NK cells, CD3-/CD56+/CD94 + NK cells, CD3-/CD56+/CD69 + NK cells, CD3-/CD56+/NKG2D + NK cells, CD3-/CD56+/NKp30 + NK cells, CD3-/CD56+/NKp46 + NK cells. Statistically significant differences *p < 0.05, **p < 0.01, ***p < 0.001, ****p < 0.00001

Similar to patients with stage IV NSCLC (*n* = 53, median 199.5 ng/mL), patients with lung metastases of different extrathoracic primary tumors (*n* = 35, median 28.6 ng/mL; *p < 0.05) (Supplementary Table 2A) also had significantly higher eHsp70 levels in the circulation than healthy controls (Fig. 4C). The follow-up of extrathoracic primary tumor patients one year after metastasectomy is shown in Supplementary Table 2B. Immunophenotyping of the peripheral blood lymphocytes by multiparameter flow cytometry revealed significantly lower lymphocyte counts (Fig. 5A) in all tumor stages and significantly decreased ratios of CD3-/CD19 + B cells in advanced tumor stages (Fig. 5B). Ratios of CD4 + T helper cells (Fig. 5C) were significantly decreased in patients with thoracic metastases compared to healthy individuals whereas those of CD8 + cytotoxic T cells were generally increased (Fig. 5C). Immunoregulatory CD4 + and CD8 + Treg cells were increased over all tumor stages (Fig. 5D). With respect to all NKT cell subtypes no significant changes to healthy controls were observed (Fig. 5E), whereas ratios of CD3-/CD56+/CD94+/NKp30+/NKp46 + NK cells but not CD3-/NKG2D + NK cells were significantly increased in patients with thoracic metastases of other primary tumors as compared to healthy controls (Fig. 5F).

### Circulating eHsp70 levels in thoracic cancer patients with and without early relapse

**Fig. 6:**
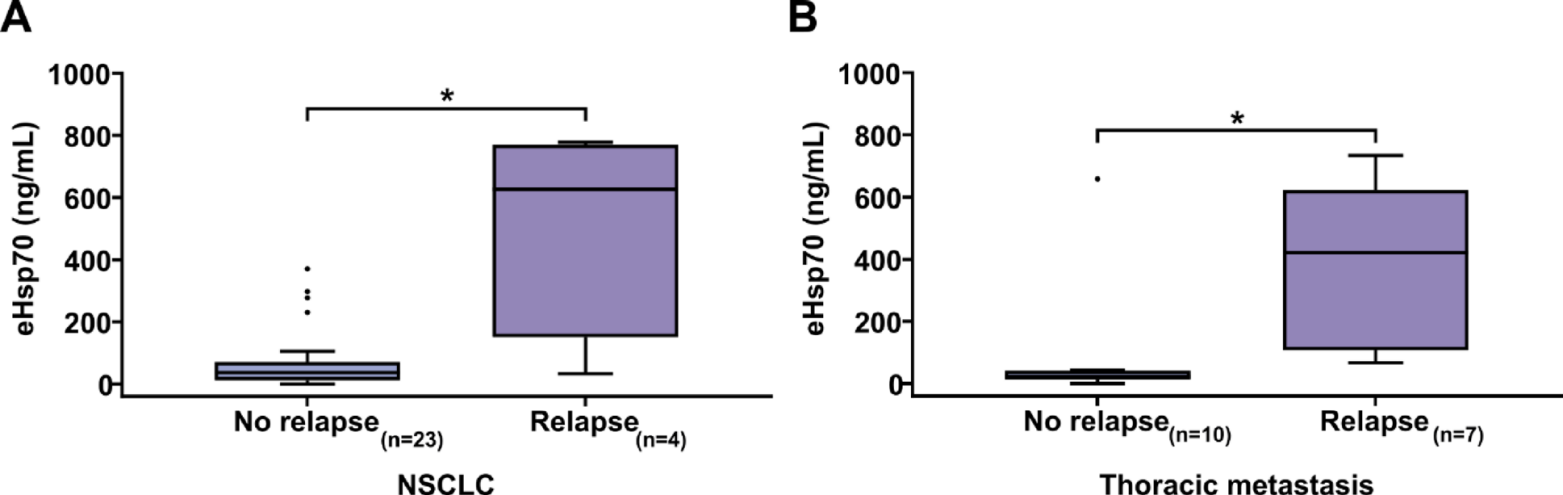
Free and vesicular eHsp70 (ng/mL) levels measured in the plasma of **A** NSCLC patients (NSCLC) and **B** patients with lung metastases (Thoracic metastases) of extrathoracic primary tumors without (No relapse) (*n* = 23, NSCLC; *n* = 10, Thoracic metastases) and with (Relapse) (*n* = 4, NSCLC 6 months; *n* = 7, Thoracic metastases 12 months) early relapse. Statistically significant differences *p < 0.05

A subgroup of NSCLC patients from whom the results of the first follow-ups were available revealed significantly higher preoperative eHsp70 values in patients with early recurrence in the first 6 months post-surgery (*n* = 4, median 626.3 ng/mL) compared to those with no evidence of recurrence in the same period (*n* = 23, median 47.5 ng/mL; *p < 0.05) (Fig. 6A). A similar observation was made in a cohort of patients with primary extrathoracic tumors following pulmonary metastasectomy with curative intent. In this group, patients with a relapse within the first postoperative year displayed significantly higher pre-operatively determined eHsp70 values (*n* = 7, median 420.8 ng/mL) compared to patients without tumor relapse (*n* = 10, median 23.3 ng/mL; *p < 0.05) (Fig. 6B).

## Discussion

In recent years, the therapeutic landscape for NSCLC has undergone rapid transformation due to a large number of innovative and well-tolerated approaches, particularly in the field of immunotherapy and targeted therapies [8, 24, 25]. These achievements have not only influenced the therapy regime for patients with advanced tumors but are also used as adjuvant and neoadjuvant approaches in early stages with favourable chances of a long-term cure [26–28]. The standard of care for localized and resectable NSCLC is now a multimodality approach which should be determined by a multidisciplinary tumor board [29]. Recently, a systematic review and meta-analysis of individual patient data from eight prospective trials of neoadjuvant or perioperative chemoimmunotherapy showed impressive event-free survival (EFS) rates of over 90% for complete pathological response and 60% for no complete pathological response at 24 months after complete resection of NSCLC [30]. These promising results underscore efforts to identify biomarkers to guide treatment intensification and de-escalation strategies. For example, in case that a blood-based biomarker could predict a complete pathological response to neoadjuvant therapy, the necessity of a planned tumor resection could be questioned or at least, it should be discussed whether the initially planned extent of resection should be limited. Efforts have been ongoing to enhance the rate of early-stage initial diagnoses, to gain insight into the tumor aggressiveness, and to identify individuals who may benefit from a more intensive therapy regime or closer follow-up care. These endeavours have led to repeated attempts to discover suitable liquid biopsy-based biomarkers having diagnostic and prognostic significance in patients with NSCLC [31, 32].

The circulating proteins CYFRA 21-1 (cytokeratin fragment 21-1) and CEA (carcinoembryonic antigen), which are among the earliest biomarker candidates for NSCLC, have been examined in several prior studies with the outcome that elevated plasma levels of these circulating proteins show a trend towards a poorer prognosis [33–38]. However, the data supporting routine measurement of these biomarkers are inconsistent, as other factors, such as inflammatory diseases and smoking, can influence the results. Therefore, neither the American [39, 40] nor the German medical societies [41] recommend routine measurement of CEA or CYFRA 21-1 for the follow-up of NSCLC patients.

Furthermore, the presence of circulating tumor cells (CTCs), which play a role in the formation and development of metastases, has been associated with poorer prognosis in patients with NSCLC [42–44]. Most recently, a large study has delivered the first promising results regarding the potential of small ribonucleic acids (RNAs) in liquid biopsies for tumor detection, albeit with a low sensitivity in early stages [45]. Despite these findings regarding suitable biomarker candidates, none of the candidates have yet been established as an integral part of the diagnostic or therapeutic process in the existing guidelines for the treatment of thoracic cancers. In the context of the upcoming introduction of lung cancer screening in Germany using LDCT, there is a significant clinical need for more specific biomarkers that could facilitate the interpretation of inconclusive results and better inform clinical decision-making.

The major goal of this study was to investigate the ability of circulating eHsp70 to predict aggressiveness and prognosis of patients with lung cancer across different tumor stages. With the established Hsp70-exo sandwich ELISA which uses the mHsp70-specific monoclonal antibodies cmHsp70.1 (aa 451–461) and cmHsp70.2 (aa 614–623), it is possible to quantify both, free Hsp70 (released predominantly by dying cells) and eHsp70 bound to lipid microvesicles (primarily derived from viable cells) in the blood [19]. The results of previous studies demonstrating significantly higher eHsp70 levels in the circulation of patients with malignant tumors compared to healthy controls [19, 23, 46] are consistent with our present findings in patients with NSCLC across all stages and patients with lung metastases of different extrathoracic primary tumors.

As a molecular chaperone, cytosolic Hsp70 maintains protein homeostasis by supporting protein folding, unfolding and transport in all nucleated cell types under physiological conditions [47]. In tumor cells, Hsp70 is frequently overexpressed and can contribute to a more aggressive and therapy-resistant tumor phenotype by interfering with apoptosis and proliferation pathways [48–50]. Many studies have shown that apart from its cytosolic localization, Hsp70 can be presented on the plasma membrane of tumor cells via a mechanism which is enabled by a tumor-specific lipid composition [51]. Furthermore, an elevated mHsp70 expression is associated with higher tumor stages and a more aggressive behaviour in various female cancers, prostate cancer and brain tumors [23, 52, 53]. Tumor cells expressing mHsp70 actively release eHsp70 in lipid microvesicles into the extracellular space [15]. Significantly increased eHsp70 levels have also been shown in patients with advanced NSCLC compared to healthy donors [46, 54]. Herein, we found a progressive increase in circulating eHsp70 levels starting from healthy donors, to low-grade NSCLC through locally advanced and metastatic NSCLC. The ROC analysis generated an AUC value of 0.83, with a specificity of 0.78 and a sensitivity of 0.73 at an eHsp70 cut-off value of 49.48 ng/mL for the presence of locally advanced or metastatic NSCLC compared to the healthy cohort. These findings further support the crucial function of eHsp70 in mediating tumor aggressiveness in lung cancer and the significant expression of Hsp70 in advanced tumors.

A second indication of Hsp70’s role as a marker for aggressiveness emerged from the detailed examination of the surgically treated cases and their histopathological results. Although some studies suggest an association between circulating eHsp70 levels and the tumor volume [55, 56], no significant association of circulating eHsp70 concentrations and the largest pathologically measured NSCLC diameter after surgical resection was found in this study. However, our results revealed a difference (***p = 0.001) in the eHsp70 concentration between patients with pathologically proven lymph node metastases and those without. In the guidelines for the pre-operative diagnosis of possible lymph node involvement and mediastinal staging in patients with NSCLC, positron emission tomography/computed tomography (PET-CT) plays a decisive role as a non-invasive method. The reported literature states that the sensitivity of PET-CT for the detection of lymph node metastases is between 0.74 and 0.85, with a specificity ranging from 0.85 to 0.92, but with low accuracy in the detection of micrometastases in affected lymph nodes [57–61]. Invasive methods such as mediastinoscopy, endobronchial ultrasound (EBUS) examination or surgical lymph node dissection can provide representative histological results [62]. To our knowledge, currently no established liquid biomarker exists for the detection of lymph node metastases in the pre-therapeutic setting. The ROC analysis of pre-operative eHsp70 in patients undergoing surgery with curative intent yielded an AUC value of 0.82 for the presence of pathological lymph node metastases with a sensitivity of 0.8 and a specificity of 0.78 at an eHsp70 value of 40.45 ng/mL. Pre-operative PET-CT results of patients with histologically confirmed lymph node metastases showed a correct clear positive finding in only 6 of 15 cases and no evidence for the presence of lymph node metastasis in 5 cases. In addition, the group with affected lymph nodes and elevated eHsp70 values exhibited a higher percentage of tumors with pathologically higher and thus more dedifferentiated grading, further suggesting a potential role of eHp70 as a marker of aggressiveness. Moreover, we could show that patients with carcinoma precursors had lower eHsp70 levels than patients with malignant tumors. In summary, we assume eHsp70 measured in the blood before start of any treatment could make a significant contribution to more precise pre-operative mediastinal staging, especially in unclear cases and in combination with unclear results in PET-CT.

In addition to NSCLC eHsp70 levels were also measured in patients with lung metastases derived from a different primary tumor than NSCLC. Like patients with locally advanced, metastatic NSCLC, these patients also exhibited significantly higher eHsp70 values compared to healthy controls, thereby indicating that eHsp70 could provide a universal tumor biomarker not only for NSCLC, but also for other tumor entities.

The third interesting evidence for the potential of eHsp70 as a tumor biomarker of aggressiveness in thoracic oncology is provided by our follow-up data. Previous studies have already shown a link between elevated eHsp70 levels and a tendency to early tumor relapse or treatment failure [23, 52, 63]. In our study, we could demonstrate in a small cohort that patients who had an early NSCLC relapse in the first 6 months showed significantly higher pre-operative eHsp70 values compared to patients without evidence of an early tumor relapse. Moreover, the eHsp70 values of 2 patients which were additionally determined at the time of the first follow-up after 3 months showed, that already at this early time point, the patient with recurrence showed a significantly higher value than the patient without recurrence. These observations align with our recently published study, wherein eHsp70 levels rose within three months post-surgery in early-relapse patients, whereas they remained stable in relapse-free patients [54].

The predictive value of eHsp70 for the occurrence of tumor relapse could also be found in patients with thoracic metastases derived form an extrathoracic primary tumor within the first 12 months following pulmonary metastasectomy. Measuring eHsp70 levels at multiple time points during follow-up and during adjuvant therapy could further confirm the results of this study and provide interesting and dynamic clues for possible recurrence and treatment response. Despite these promising results further studies with larger patient cohorts are necessary to verify the value of Hsp70 as a diagnostic biomarker.

The immunophenotyping of the peripheral blood lymphocytes of patients with lung cancer did not provide conclusive results as a predictive tumor biomarker for tumor aggressiveness. Irrespective of the stage and tumor aggressiveness all patients suffered from a lymphopenia and the numbers of immunosuppressive Treg cells were found to be significantly increased compared to healthy controls. An increase in CD3-/CD56+/CD94+/CD69+/NKp30+/NKp46 + NK cell subsets but not CD3-/CD56+/NKG2D + NK cells which correlated with a significant drop in CD4 + T helper cells, appeared to be restricted to patients with lung metastases of an extrathoracic primary tumor. Since reduced CD4 + T cell counts are associated with decreased levels of pro-inflammatory cytokines such as IL-2 in the circulation [43], we assume that despite an increase in some NK cell subsets, these effector cells are unable to mediate protective anti-tumor immunity in advanced tumors. Previous work of our group indicated that Hsp70 in the presence of IL-2 can stimulate the proliferative and cytotoxic activity of NK cells [64]. In the absence of IL-2 Hsp70 cannot stimulate NK cell activity. Moreover, increased proportions of Treg cells promote immunosuppression of NK cells in patients with thoracic metastases.

Since we found no obvious association between circulation eHsp70 levels and tumor size, we hypothesize that the tumor aggressiveness (lymph node metastases, early relapse), rather than the size of the tumor, is responsible for the elevated circulating eHsp70 levels. Considering the increased detection of incidental pulmonary nodules by lung cancer screening, our easily measurable biomarker in liquid biopsies could contribute to the management of such unclear cases. eHsp70 values might be superior to other soluble markers such as CEA or CYFRA 21 − 1 that are affected by inflammatory diseases and smoking [39, 40]. However, CEA and CYFRA 21 − 1 were not measured in our patient cohort and therefore a direct comparison of the biomarkers was not possible. In instances where elevated eHsp70 values are associated with ambiguous results, early histological confirmation or closer follow-up could be considered. On the other hand, measuring eHsp70 not only at the time of diagnosis, but also during the treatment could help to identify patients experiencing treatment failure and thus guide the selection of alternative treatment strategies or more intensive approaches. This is especially relevant given the increasing complexity and diversity of available modalities.

Despite the encouraging findings, this prospective study has some limitations that should be acknowledged when interpreting the results. First, the sample size was relatively small, which limits the statistical power and generalizability of the findings. While some significant differences were observed, smaller effects may have gone undetected due to insufficient power. In addition, the possibility of residual confounding from unmeasured factors cannot be entirely excluded. The limitations are primarily due to the exploratory nature of this pilot study and its complex immunological work. Therefore, future studies with larger, adequately powered, prospectively matched cohorts are warranted in order to validate and expand upon these results.

## Conclusions

With the imminent launch of a national lung cancer screening initiative in Germany and the high number of incidental pulmonary nodules on LDCT, there is an urgent need for reliable biomarkers to guide subsequent diagnostic algorithms. Our data support eHsp70 as an indicator of disease aggressiveness in thoracic cancers. In particular, we observed a consistent and significant increase in eHsp70 levels with advancing tumor stages. Patients with lymph node metastases and de-differentiated tumors had significantly higher eHsp70 levels compared to patients without such disease characteristics. In addition, the association between elevated pre-operative eHsp70 levels and early tumor recurrence underscores its prognostic value. However, further studies are warranted to validate our findings in larger patient cohorts.

## Electronic supplementary material

Below is the link to the electronic supplementary material.

Supplementary Material 1. Supplementary Figure 1: Proportions of lymphocyte subsets in the peripheral blood of healthy donors and NSCLC patients with adeno and squamous cell carcinoma histology. (A) lymphocytes, (B) CD3-/CD19+ B cells, (C) CD3+ T cells, (D) CD3+/CD4+ T helper cells, (E) CD3+/CD8+ cytotoxic T cells, (F) CD3+/CD4+/FoxP3+ Treg cells, (G) CD3+/CD8+/FoxP3+ Treg cells, (H) CD3+/CD56+ NKT cells, (I) CD3+/CD94+/CD56+ NKT cells, (J) CD3+/CD69+/CD56+ NKT cells, (K) CD3+/NKG2D+/CD56+ NKT cells, (L) CD3-/CD56+ NK cells, (M) CD3-/CD94+ NK cells, (N) CD3-/CD69+/CD56+ NK cells, (O) CD3-/NKG2D+ NK cells, (P) CD3-/NKp30+ NK cells, (Q) CD3-/NKp46+ NK cells. N numbers of samples (n) are indicated in each graph, statistically significant differences *p<0.05, **p<0.01, ***p<0.001, ****p<0.00001

Supplementary Material 2. Supplementary Figure 2: Free and vesicular eHsp70 (ng/mL) levels measured in the plasma of healthy individuals (n=108) and NSCLC patients in the respective tumor stages (A): I (n=25), II (n=11), III (n=89) and IV (n=53) or (B) IA (n=16), IB (n=9), IIB (n=11), IIIA (n=49), IIIB (n=40), IV (n=53). Statistically significant differences *p<0.05, **p<0.01, ***p<0.001

Supplementary Material 3. Supplementary Figure 3: Correlative analysis of the largest tumor diameter (cm) of the surgically resected tumors and eHsp70 levels (ng/mL) in NSCLC patients (n=47, R2=0.0616, mean diameter 3.26 cm)

Supplementary Material 4

Supplementary Material 5

## Data Availability

The datasets used and/or analyzed during the current study are available from the corresponding author on reasonable request.

## Abbreviations

AUC: Area under the curve
APC: Allophycocyanine
CEA: Carcinoembryonic antigen
CYFRA: Cytokeratin fragment
CTC: Circulating tumor cell
EBUS: Endobronchial ultrasound
EDTA: Ethylenediamine tetraacetic acid
eHsp70: Extracellular heat shock protein 70
ELISA: Enyzme-linked immunosorbent assay
EVS: Event-free survival
FITC: Fluorescein isothiocyanate (525 nm emission)
Hsp70: Heat shock protein 70 (new nomenclature HSPA1A)
HRP: Horseradish peroxidase
LDCT: Low-dose computed tomography
mHsp70: Membrane-expressed Hsp70
NK cell: Natural killer cell
NKT cell: Natural killer-like T cell
NSCLC: Non-small cell lung cancer
PD-L1: Programmed cell death protein 1 ligand
PE: Phycoerytherin (578 nm emission)
PerCP: Peridinin chlorophyll A protein
PET-CT: Positron emission tomography/computed tomography
RNA: Ribonucleic acid
ROC: Receiver operating characteristic
Treg: Immunoregulatory T cell
UICC: Union for International Cancer Control
