# Supplementary material for: Circulating Hsp70: a tumor biomarker for lymph node metastases and early relapse in thoracic cancer
Source: BMC Cancer. 2025 Aug 9;25:1297. doi: 10.1186/s12885-025-14725-5 (PMC12335804; doi:10.1186/s12885-025-14725-5)
Supplement: Supplementary file 1 — Supplementary Material 1. Supplementary Figure 1: Proportions of lymphocyte subsets in the peripheral blood of healthy donors and NSCLC patients with adeno and squamous cell carcinoma histology. (A) lymphocytes, (B) CD3-/CD19+ B cells, (C) CD3+ T cells, (D) CD3+/CD4+ T helper cells, (E) CD3+/CD8+ cytotoxic T cells, (F) CD3+/CD4+/FoxP3+ Treg cells, (G) CD3+/CD8+/FoxP3+ Treg cells, (H) CD3+/CD56+ NKT cells, (I) CD3+/CD94+/CD56+ NKT cells, (J) CD3+/CD69+/CD56+ NKT cells, (K) CD3+/NKG2D+/CD56+ NKT cells, (L) CD3-/CD56+ NK cells, (M) CD3-/CD94+ NK cells, (N) CD3-/CD69+/CD56+ NK cells, (O) CD3-/NKG2D+ NK cells, (P) CD3-/NKp30+ NK cells, (Q) CD3-/NKp46+ NK cells. N numbers of samples (n) are indicated in each graph, statistically significant differences *p<0.05, **p<0.01, ***p<0.001, ****p<0.00001 [file 12885_2025_14725_MOESM1_ESM.docx]

A B

C D E

Supplementary Figure 1

F G

Supplementary Figure 1

H I


J K

Supplementary Figure 1

L M

N O

Supplementary Figure 1

P Q

Supplementary Figure 1
