# Supplementary figures and images for: Circulating Hsp70: a tumor biomarker for lymph node metastases and early relapse in thoracic cancer
Source: BMC Cancer. 2025 Aug 9;25:1297. doi: 10.1186/s12885-025-14725-5 (PMC12335804; doi:10.1186/s12885-025-14725-5)

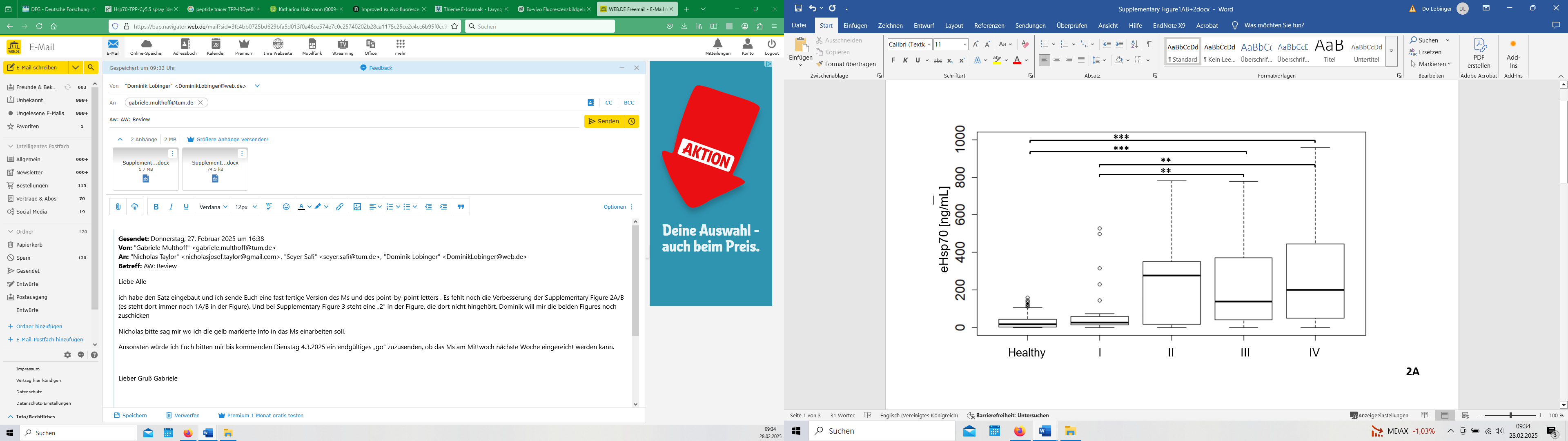


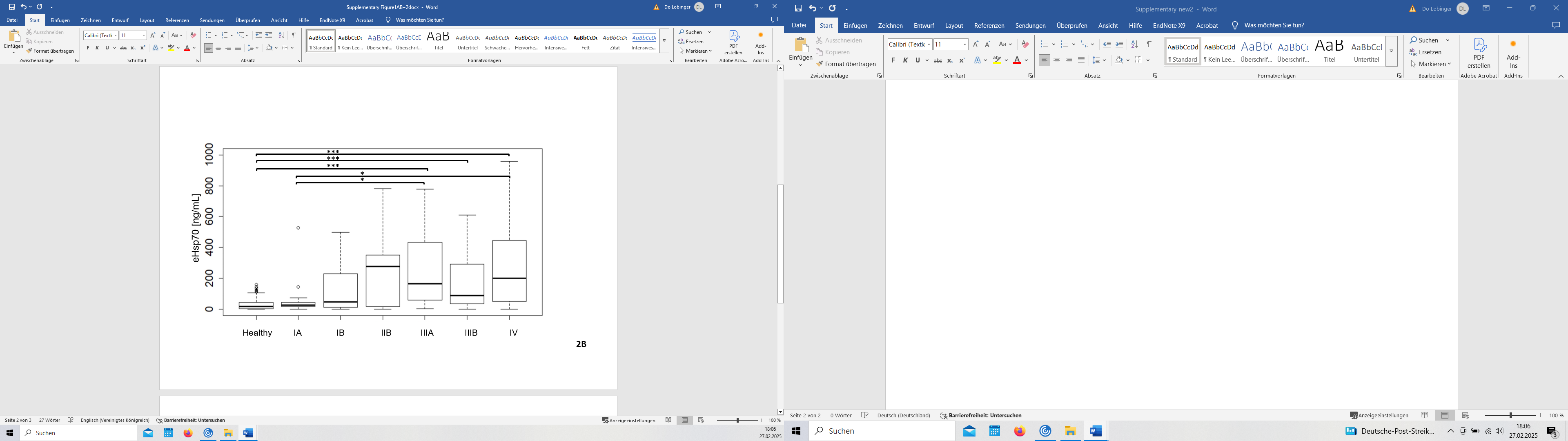

Supplement: Supplementary file 2 — Supplementary Material 2. Supplementary Figure 2: Free and vesicular eHsp70 (ng/mL) levels measured in the plasma of healthy individuals (n=108) and NSCLC patients in the respective tumor stages (A): I (n=25), II (n=11), III (n=89) and IV (n=53) or (B) IA (n=16), IB (n=9), IIB (n=11), IIIA (n=49), IIIB (n=40), IV (n=53). Statistically significant differences *p<0.05, **p<0.01, ***p<0.001 [file 12885_2025_14725_MOESM2_ESM.docx]
