# Supplementary material for: Circulating Hsp70: a tumor biomarker for lymph node metastases and early relapse in thoracic cancer
Source: BMC Cancer. 2025 Aug 9;25:1297. doi: 10.1186/s12885-025-14725-5 (PMC12335804; doi:10.1186/s12885-025-14725-5)
Supplement: Supplementary file 4 — Supplementary Material 4 [file 12885_2025_14725_MOESM4_ESM.docx]

Supplementary Table 1A: Patient characteristics of surgical cases

| Stage | ∑n |  | n | Adeno | SCC | Other | Median age | Female | Male |
| --- | --- | --- | --- | --- | --- | --- | --- | --- | --- |
| I | 25 | *IA* | *16* | 17 | 5 | 3 | 71 | 15 | 10 |
|  |  | *IB* | *9* |  |  |  |  |  |  |
| II | 11 | *IIA* | */* | 5 | 1 | 5 | 73 | 4 | 7 |
|  |  | *IIB* | *11* |  |  |  |  |  |  |
| III | 17 | *IIIA* | *15* | 7 | 8 | 2 | 64 | 4 | 13 |
|  |  | *IIIB* | *2* |  |  |  |  |  |  |

Supplementary Table 1B: TNM, UICC-classification (WHO 2017) of surgical cases

| Stage, TNM, UICC  (WHO 2017) | | |  |  |  |
| --- | --- | --- | --- | --- | --- |
|  | | |  |  |  |
|  | n=25 |  | n=11 |  | n=17 |
| IA | pT1c, pN0 | IIB | pT3, pN0 | IIIA | pT3, pN1 |
|  | pT1c, pN0 |  | pT3, pN0 |  | pT4, pN0 |
|  | pT1c, pN0 |  | pT3, pN0 |  | pT1b, pN2 |
|  | pT1c, pN0 |  | pT1c, pN1 |  | pT3, pN1 |
|  | pT1c, pN0 |  | pT3, pN0 |  | T2a, pN2 |
|  | pT1b, pN0 |  | pT2, pN1 |  | pT1c, pN2 |
|  | pT1c, pN0 |  | pT2a, pN1 |  | pT4, pN0 |
|  | pT1b, pNX |  | pT2a, pN1 |  | pT4, pN0 |
|  | pT1b, pN0 |  | PT3, pN0 |  | pT2a, pN2 |
|  | pT1b, pN0 |  | PT2a, pN1 |  | pT3, pN1 |
|  | pT1c, pN0 |  | PT3, pN0 |  | pT4, pN0 |
|  | pT1c, pN0 |  |  |  | pT2b, pN2 |
|  | pT1c, pN0 |  |  |  | pT4, pN0 |
|  | pT1c, pN0 |  |  |  | pT4, pN0 |
|  | pT1b, pN0 |  |  |  | pT4, pN0 |
|  | PT1a, pN0 |  |  |  |  |
|  |  |  |  | IIIB | pT4, pN2 |
| IB | pT2a, pN0 |  |  |  | pT4, pN2 |
|  | pT2a, pN0 |  |  |  |  |
|  | pT2a, pN0 |  |  |  |  |
|  | pT2a, pN0 |  |  |  |  |
|  | pT2a, pN0 |  |  |  |  |
|  | pT2a, pNX |  |  |  |  |
|  | pT2a, pN0 |  |  |  |  |
|  | pT2b, pN0 |  |  |  |  |
|  | pT2a, pN0 |  |  |  |  |
