# Supplementary material for: Circulating Hsp70: a tumor biomarker for lymph node metastases and early relapse in thoracic cancer
Source: BMC Cancer. 2025 Aug 9;25:1297. doi: 10.1186/s12885-025-14725-5 (PMC12335804; doi:10.1186/s12885-025-14725-5)
Supplement: Supplementary file 5 — Supplementary Material 5 [file 12885_2025_14725_MOESM5_ESM.docx]

Supplementary Table 2A: Entity of extrathoracic primary tumors

| Tumor entity | n |
| --- | --- |
| Sarcoma | 3 |
| Testicular carcinoma | 6 |
| Colorectal cancer | 8 |
| Melanoma | 2 |
| Breast cancer | 1 |
| Renal cell carcinoma | 2 |
| Pharyngeal carcinoma | 3 |
| Pancreatic carcinoma | 1 |
| Thymus carcinoma | 1 |
| Squamous cell carcinoma | 1 |
| Prostate cancer | 4 |
| Urothelial carcinoma | 3 |
| **∑** | 35 |
| Female | 9 |
| Male | 26 |
| Median age [years] | 64 |

Supplementary Table 2B: Follow-up of extrathoracic primary tumors (1 year after metastasectomy)

| Without relapse | | n |  | Relapse |  | n |
| --- | --- | --- | --- | --- | --- | --- |
|  |  |  |  |  |  |  |
| Pharyngeal carcinoma | | 2 |  | Urothelial carcinoma | | 3 |
| Sarcoma |  | 2 |  | Colorectal cancer | | 2 |
| Testicular carcinoma | | 2 |  | Thymus carcinoma | | 1 |
| Squamous cell carcinoma | | 1 |  | Pancreatic carcinoma | | 1 |
| Prostate cancer | | 1 |  |  |  |  |
| Colorectal cancer | | 1 |  |  |  |  |
| Melanoma |  | 1 |  |  |  |  |
