# Supplementary material for: Circulating Hsp70: a tumor biomarker for lymph node metastases and early relapse in thoracic cancer
Source: BMC Cancer. 2025 Aug 9;25:1297. doi: 10.1186/s12885-025-14725-5 (PMC12335804; doi:10.1186/s12885-025-14725-5)
Supplement: Supplementary file 5 [file 12885_2025_14725_MOESM3_ESM.docx]

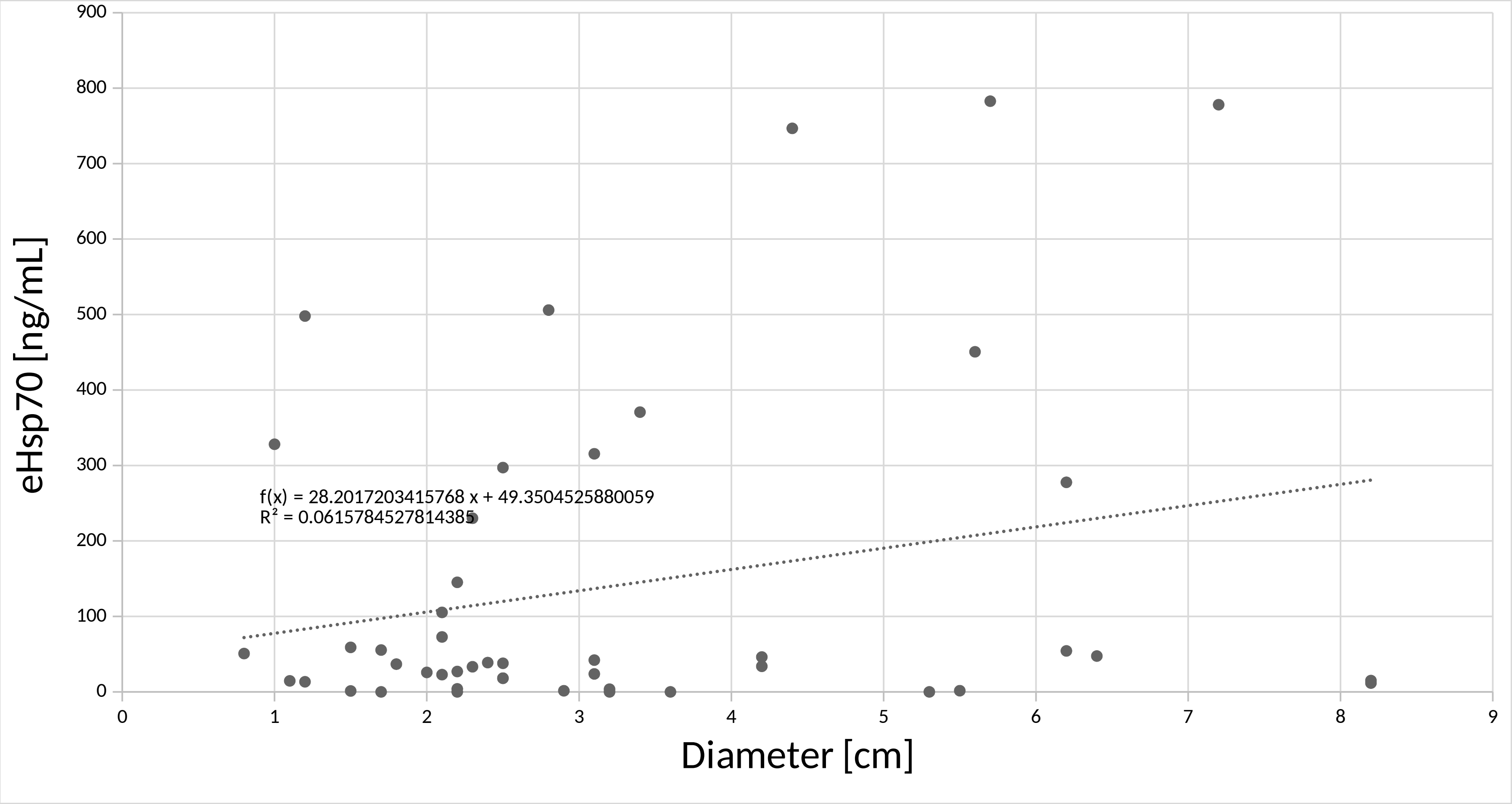
eHsp70 [ng/mL]
900
800
700
600
500
400
300
200
100
0
0
1
2
3
4
5
6
7
8
9
Diameter [cm]
f(x) = 28.2017203415768 x + 49.3504525880059
R² = 0.0615784527814385
